# Survival in Hostile Conditions: Pupylation and the Proteasome in Actinobacterial Stress Response Pathways

**DOI:** 10.3389/fmolb.2021.685757

**Published:** 2021-06-07

**Authors:** Tatjana von Rosen, Lena ML Keller, Eilika Weber-Ban

**Affiliations:** Institute of Molecular Biology and Biophysics, ETH Zurich, Zurich, Switzerland

**Keywords:** pupylation, bacterial proteasome, degradation, DNA Damage, metal homeostasis, oxidative stress, mycobacteria, *Mycobacterium tuberculosis*

## Abstract

Bacteria employ a multitude of strategies to cope with the challenges they face in their natural surroundings, be it as pathogens, commensals or free-living species in rapidly changing environments like soil. Mycobacteria and other Actinobacteria acquired proteasomal genes and evolved a post-translational, ubiquitin-like modification pathway called pupylation to support their survival under rapidly changing conditions and under stress. The proteasomal 20S core particle (20S CP) interacts with ring-shaped activators like the hexameric ATPase Mpa that recruits pupylated substrates. The proteasomal subunits, Mpa and pupylation enzymes are encoded in the so-called Pup-proteasome system (PPS) gene locus. Genes in this locus become vital for bacteria to survive during periods of stress. In the successful human pathogen *Mycobacterium tuberculosis*, the 20S CP is essential for survival in host macrophages. Other members of the PPS and proteasomal interactors are crucial for cellular homeostasis, for example during the DNA damage response, iron and copper regulation, and heat shock. The multiple pathways that the proteasome is involved in during different stress responses suggest that the PPS plays a vital role in bacterial protein quality control and adaptation to diverse challenging environments.

## Introduction

**FIGURE 1:**
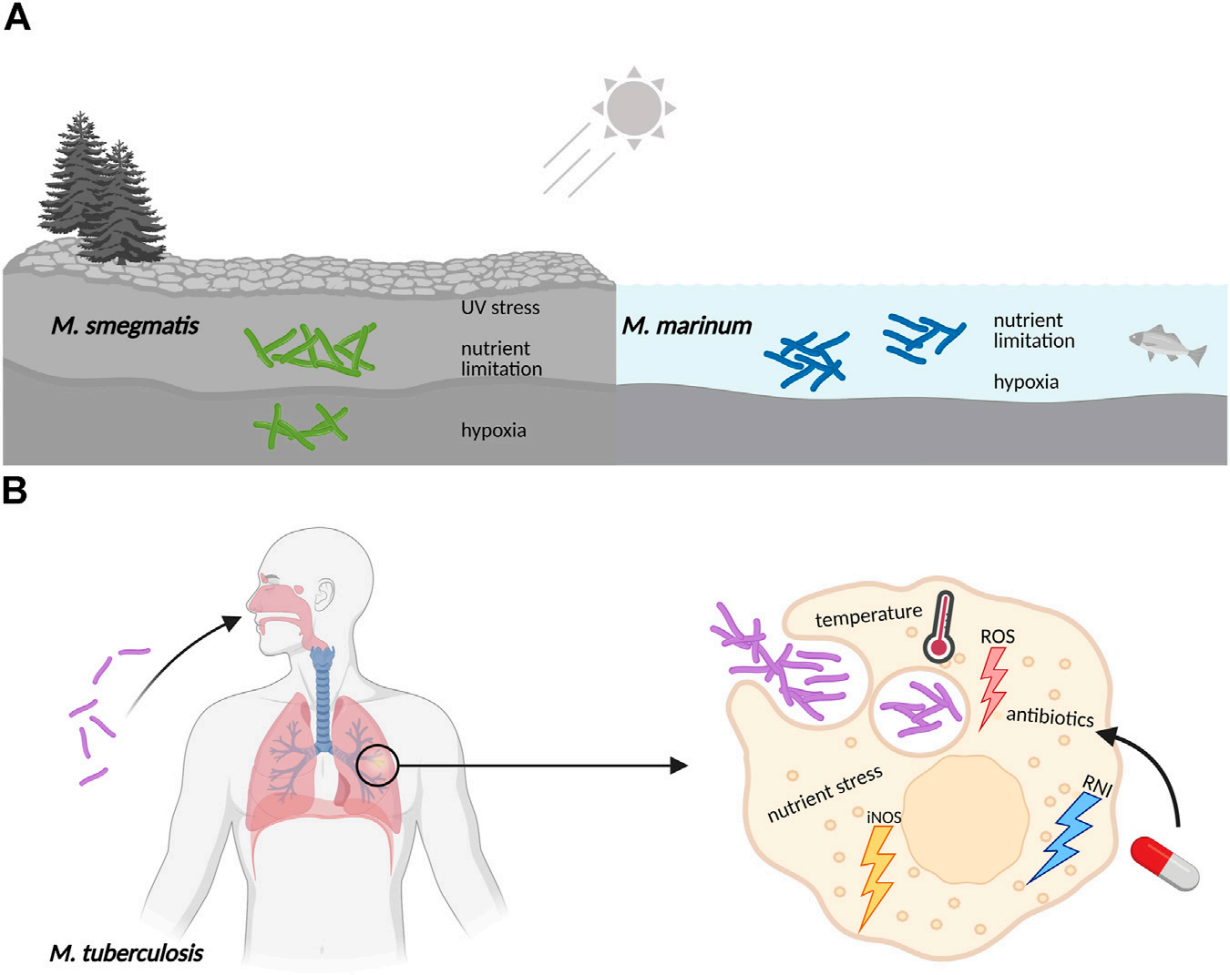
Mycobacteria frequently encounter adverse conditions in their natural surroundings, requiring coordinated stress responses. **(A)** Free living mycobacteria, like the soil-dwelling *M. smegmatis* or the ocean bacterium *M. marinum*, are exposed to UV radiation, experience sudden changes in temperature, have to adjust to varying oxygen levels, and need to survive periods of limitation in essential nutrients like carbon, nitrogen and iron minerals. **(B)** Pathogenic mycobacteria like *M. tuberculosis* are exposed to hostile environments when growing inside host macrophages, where they also face sudden changes in nutrient availability, pH and temperature. The defensive mechanisms of macrophages include the production of reactive oxygen species (ROS), nitric oxide synthase in its inducible isoform (iNOS), and reactive nitrogen intermediates (RNI). Furthermore, exposure to antibiotics during drug treatment of *M. tuberculosis* infections also elicits stress responses in an attempt to evade killing and develop resistance.

Bacteria cultured in the laboratory are generally grown in pure, liquid culture under optimal conditions. They are provided with a balanced mix of nutrients including carbon and nitrogen sources along with minerals and trace elements, shaken for good aeration and kept at their favorite temperature. However, in the real world, bacterial life is frequently harsh and far from ideal (Haruta and Kanno, 2015). This certainly applies to Actinobacteria that constitute one of the largest and most diverse phyla in the bacterial kingdom (Barka et al., 2016). Its members exhibit a variety of lifestyles: as soil inhabitants living in rapidly changing environments in competition or symbiosis with other microorganisms, as pathogens under nutrient limitation and subject to host defense mechanisms or as plant and gastrointestinal commensals. As a consequence of their exposure to changing nutritional conditions and a multitude of stresses, Actinobacteria evolved a particularly high adaptive ability allowing them to persist in their often-hostile environments (Figure 1).

Adaptation ultimately requires that the proteome present at a given moment under a defined set of conditions can be reshaped efficiently to reflect the new needs of the organism. On the one hand this is achieved through changes in gene expression involving regulation on the transcriptional and translational levels (Guo and Gross, 2014; Martin and Liras, 2020). On the other hand, an efficient and robust proteomic response to stress and changing nutritional states also requires controlled protein turnover (Gerth et al., 2008; Michalik et al., 2012; Guo and Gross, 2014). The balanced production and breakdown of proteins referred to collectively as protein homeostasis, is also required under normal conditions and presents a fundamental activity of all living cells. The degradation branch of protein homeostasis permits a rapid adaptive response that is independent of and complements the changes in transcription and translation. It is also independent of bacterial growth and therefore particularly important for bacteria that are not growing actively (Trotschel et al., 2013), such as pathogenic bacteria residing in the host in dormant or extremely slow-growing states or bacteria under nutrient limiting conditions.

Mycobacteria are amongst the most notorious members of Actinobacteria, owed largely to *Mycobacterium tuberculosis* (Mtb), one of the most successful human pathogens of all time, currently responsible for 1.5 million deaths and more than 10 million infections every year worldwide (WHO, 2020). As an intracellular pathogen, Mtb has to survive behind enemy lines, residing inside host macrophages, where it needs to resist the defensive onslaught of oxidative and nitrosative stress and adapt to nutritional deficiencies (Awuh and Flo, 2017). Mtb can persist in the host in a slow-growing/dormant state for decades, from which it can resume growth and progress to clinical disease (Jayachandran et al., 2012). The identification of pathways supporting persistence in the host has long been a focus of research toward combatting Mtb. One such pathway is centered around a bacterial proteasome gene locus characteristic to the phylum of Actinobacteria (Darwin et al., 2003; Gandotra et al., 2007). The existence of proteasomes in this phylum has been known since the early 1990’s, when a proteasomal particle was first observed in nitrogen-fixing bacterium Frankia (Benoist et al., 1992). However, unlike eukaryotes, bacteria generally do not encode proteasomal subunits, but have their own version of compartmentalizing proteases (Clp proteases, HslUV and FtsH) that are responsible for regulated protein turnover and protein quality control (Knipfer et al., 1999; Laederach et al., 2014). The proteasome is thus an unusual occurrence in bacteria restricted largely to the phylum Actinobacteria, where it is found in addition to other typical bacterial degradation complexes (Laederach et al., 2014). Originally adopted by horizontal gene transfer, the proteasome gene locus has evolved to support the organisms during stress, for example in Mtb contributing to its persistence inside host macrophages (Darwin et al., 2003; Gandotra et al., 2007).

**FIGURE 2:**
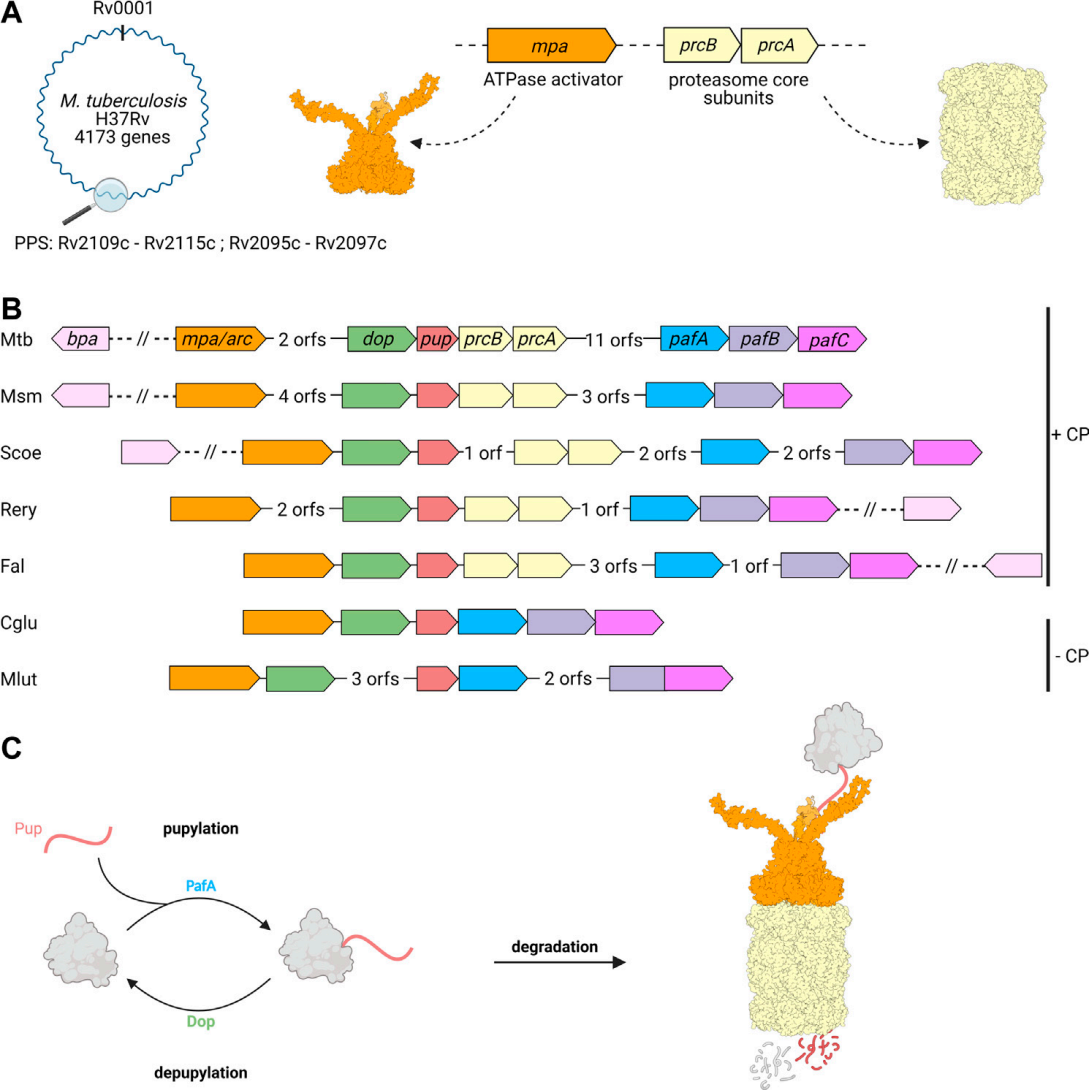
Genes involved in pupylation and Pup-dependent proteasomal degradation are organized in the Pup-proteasome gene locus in Actinobacteria. **(A)** Members of the Pup-proteasome system (PPS) are encoded in close proximity to one another in the PPS gene locus, ranging from Rv2109c to Rv 2097c in Mtb. The genes coding for the hexameric ATPase Mpa (Rv2115c, orange) and proteasomal α and β subunits PrcA and PrcB (Rv2109 and Rv2110c, beige), were most likely acquired by horizontal gene transfer. **(B)** In addition to Mpa/ARC and the 20S proteasomal subunits, the PPS gene locus also encodes the ubiquitin-like protein Pup (Rv2111c, red), the Pup-ligase PafA (Rv 2097c, blue), and the depupylase Dop (Rv2112c, green). The transcriptional regulator PafBC (Rv 2096c and Rv 2095c, purple), is found in close proximity to PafA and has been shown to regulate the major DNA damage response in mycobacteria, to which the PPS is tightly linked. The alternative ring shaped ATP- and pupylation-independent activator Bpa (Rv3780, light pink) is not found in the Pup-Proteasome gene locus, but strictly co-occurs with the proteasomal subunit genes across Actinobacteria. A subgroup of Actinobacteria is missing the proteasomal subunit genes in their PPS locus, likely by secondary loss during evolution. Mtb, *Mycobacterium tuberculosis*; Msm, *Mycobacterium smegmatis*; Scoe, *Streptomyces coelicolor*; Rery, *Rhodococcus erythropolis*; Fal, *Frankia alni*; Cglu, *Corynebacterium glutamicum*; Mlut, *Micrococcus luteus*. **(C)** Pupylation involves the post-translational modification of target proteins (grey) with Pup (red), and is catalyzed by the Pup ligase PafA. Pupylated proteins are either depupylated by the deamidase Dop or recruited to the Mpa-20S proteasome complex for unfolding and degradation.

Around the 20S CP and its regulatory ATPase partner Mpa, both homologous to eukaryotic 26S proteasomes, a novel substrate recruitment pathway has evolved in Actinobacteria that shows functional parallels to ubiquitination, but is of distinct, exclusively bacterial origin (Pearce et al., 2008; Burns et al., 2009; Sutter et al., 2009). Through this pathway termed pupylation, substrate proteins are post-translationally modified with the small, intrinsically disordered protein Pup (prokaryotic ubiquitin-like protein) (Pearce et al., 2006; Burns et al., 2009). Pupylation can be reversed and both the ligase and depupylase enzymes are encoded in the gene locus where the genes for the proteasomal degradation machinery and the modifier Pup reside (Burns et al., 2010; Imkamp et al., 2010). This locus, referred to as the Pup proteasome system (PPS) gene locus, is present in all Actinobacteria, however, a subset of organisms in this phylum has lost the proteasomal subunits despite maintaining pupylation (Figure 2).

In this review article, we highlight the roles that pupylation and the Pup proteasome gene locus play in the survival strategy of mycobacteria and other Actinobacteria under stress.

## Pupylation-Dependent Proteasomal Degradation Plays a Role in Actinobacterial Stress Responses

The actinobacterial 20S proteasome, like its eukaryotic relative, forms the core of the fully assembled protease complex (Nagy et al., 1997; Wolf et al., 1998; Lin et al., 2006). It is built from four stacked homo-heptameric rings, the inner two β-rings carrying the proteolytic active sites, framed by the two α-rings that shape the entrance pores into the degradation chamber. In order to degrade substrate proteins post-translationally modified with Pup, the α-rings of the 20S particle associate with a likewise ring-shaped hexameric ATPase of the AAA family, called Mpa (mycobacterial proteasome ATPase) in mycobacteria or ARC (ATPase forming ring-shaped complexes) in other Actinobacteria (Wolf et al., 1998; Darwin et al., 2005; Striebel et al., 2010). Mpa employs a C-terminal proteasome interaction motif (GQYL) that retains the same penultimate aromatic residue as the HbYX motif of eukaryotic proteasome interactors (Darwin et al., 2005; Pearce et al., 2006; Smith et al., 2007; Rabl et al., 2008; Striebel et al., 2010). Both motifs insert into deep binding pockets located between the α-subunits of the 20S CP, featuring a binding site for the aromatic residue and a lysine residue to interact with the C-terminal carboxylate.

The covalent modification of proteins at lysine residues with Pup is catalyzed by the ligase PafA (proteasome accessory factor A) in a two-step mechanism (Guth et al., 2011). This involves the formation of an isopeptide bond between the C-terminal glutamate side chain carboxylate of Pup and the ε-amino group of the target lysine by nucleophilic substitution. PafA has been suggested to have evolved from an ancient glutamyl-amine ligase enzyme (glutamine synthetase or γ-glutamyl cysteine ligase) based on its sequence and structural homology to this class of enzymes (Iyer et al., 2008; Sutter et al., 2009; Özcelik et al., 2012; Hecht et al., 2021). The structurally homologous enzyme Dop (deamidase of Pup), also encoded in the PPS gene locus, catalyzes the opposing activity by cleaving the isopeptide bond between Pup and substrates (Burns et al., 2010; Cerda-Maira et al., 2010; Imkamp et al., 2010; Bolten et al., 2017). Mycobacteria and a number of other Actinobacteria encode Pup with a C-terminal glutamine instead of glutamate, necessitating deamidation of the glutamine side chain prior to ligation. Interestingly, deamidation, which is chemically equivalent to depupylation, is also carried out by Dop (Striebel et al., 2009).

The modifier Pup despite its name is unrelated in structure and sequence to ubiquitin. Pup is an intrinsically disordered protein of 64 residues with a molecular mass under 7 kDa in Mtb (Chen et al., 2009; Liao et al., 2009; Sutter et al., 2009). The disordered ensemble state typical for intrinsically disordered proteins is present in both free Pup and when it is covalently attached to protein substrates (Barandun et al., 2017). However, it undergoes disorder-to-order transitions upon binding to the pupylation enzymes (ligase and depupylase) (Barandun et al., 2013) or to the proteasomal regulator Mpa (Wang et al., 2010). Interestingly, it adopts different conformations depending on the binding partner (Delley et al., 2017). Interaction with the ligase and depupylase results in the formation of two orthogonal, shorter helices that ensure a snug fit into the Pup binding groove of the enzymes (Barandun et al., 2013). As a recognition tag for proteasomal degradation, Pup binds to the N-terminal coiled-coil domains at the Mpa ring surface, forming a single longer helix that joins the coiled-coil (Sutter et al., 2009; Wang et al., 2010). Once bound, Mpa/ARC unfolds the pupylated substrate and translocates it into the 20S proteasome degradation chamber in an ATP-dependent manner (Striebel et al., 2010).

In addition to the genes required for pupylation and proteasomal degradation, including *pup*, *pafA*, *dop*, *arc/mpa*, and the proteasomal subunits *prcA/B*, the PPS gene locus also encodes the transcriptional regulator PafBC (Olivencia et al., 2017) (Figure 2B). In mycobacteria, PafBC (proteasome accessory factor B and C) is encoded in the same operon together with the Pup ligase PafA, giving rise to the name (Festa et al., 2007). Although this is not the case in all Actinobacteria, the *pafBC* genes are nevertheless tightly associated with the PPS locus and are found in close proximity downstream of the *pafA* gene. PafBC is important for the mycobacterial DNA damage response and ties the PPS locus to stress conditions with DNA damaging potential like oxidative stress, UV exposure or DNA damaging natural compounds produced by other microorganisms (Müller et al., 2018).

Besides Mpa/ARC, the 20S bacterial proteasome can interact with alternative ring-shaped activators not encoded in the Pup proteasome gene locus, including the ATPase Cpa (Cdc48-like protein of Actinobacteria) and the ATP-independent Bpa (bacterial proteasome activator, also referred to as PafE) (Delley et al., 2014; Jastrab et al., 2015; Ziemski et al., 2018). Although recruitment of substrate proteins to these alternative proteasomal complexes does not involve pupylation, they also play a role in adaptation to stressful conditions and will be discussed later in this review.

### Mycobacterial Stress Responses to Nitrogen Starvation or Reactive Nitrogen Intermediates Involve Pupylation and Proteasomal Degradation

Nitrogen plays an essential role for all living organisms, since it is a major constituent of the biological molecules making up a cell’s proteome, its hereditary material in the form of DNA and RNA, signaling molecules, cell wall constituents, cofactors and many other fundamentally important biomolecules. Bacteria generally use it in its reduced form as ammonium for incorporation into amino acids, thereby providing the building blocks for protein biogenesis. This involves uptake via ammonium transporters and assimilation into glutamine or glutamate via glutamine synthetase and glutamate synthase or glutamate dehydrogenase (Herrero et al., 2019). Of course, bacteria can also directly take up glutamate and glutamine or other amino acids like arginine and aspartate that can be further metabolized. In fact, Mtb is able to take up all 20 proteinogenic amino acids from the environment and prefers amino acids over ammonia as nitrogen source (Agapova et al., 2019). Furthermore, when ammonium is scarce, Actinobacteria, like most bacteria can take up nitrogen from the environment in the form of nitrate and metabolize it to ammonium. In this pathway, nitrate is first metabolized to nitrite *via* the nitrate reductase NarGHIJ complex, and the nitrite reductase complex NirBD then further reduces nitrite to ammonium that can be assimilated into amino acids (Malm et al., 2009). These enzymes contain iron-sulfur clusters and the reductions involve radical chemistry.

Regulatory mechanisms of nitrogen metabolism are geared toward ensuring sufficient nitrogen supply, and they generate a swift response to changed nutritional conditions like different nitrogen sources, nitrogen limitation or starvation. At the same time, the organism must avoid accumulation of toxic nitrogen compounds like nitrite that have the ability to produce radicals and damage DNA, lipids and proteins. Furthermore, pathogenic organisms have to contend with reactive nitrogen species generated by host defense mechanisms. Consequently, Actinobacteria can experience stress connected to nitrogen metabolism in two ways, as starvation stress or as toxic stress. The PPS appears to play a role in both types of nitrogen stress.

Mtb is an intracellular pathogen that makes a home of the very cells that phagocytose it (Huang et al., 2019). Inside the host macrophage, Mtb prevents phagosome maturation and ultimately persists in this organelle. During establishment of persistence, the interplay of host immune defense mechanisms and Mtb evasive counteraction results in formation of a granuloma, a walled-off, fibrous structure with a macrophage-rich center, where Mtb reside and slowly proliferate (Queval et al., 2017; BoseDasgupta and Pieters, 2018). An important factor in controlling Mtb infection is the production of nitric oxide by activated host macrophages through the activity of inducible nitric oxide synthase (NOS2) (MacMicking et al., 1997) as well as reactive oxygen species (ROS) via the superoxide generating enzyme NOX2 (Fang, 2004). Inside the phagosome, this leads to the generation of nitrite that can be protonated and produce radical forms of nitric oxide and other lethal nitrogen intermediates (Stuehr and Nathan, 1989). A transposon mutagenesis screen aimed at identifying genes that render Mtb more resistant to reactive nitrogen intermediates uncovered a role of the proteasome gene locus in survival of nitrosative stress (Darwin et al., 2003). Disruption of both *mpa* and *pafA* resulted in increased sensitivity of Mtb to acidified nitrite and cultures treated with proteasome inhibitor also showed less resistance under these conditions. Furthermore, the deletion strains were attenuated in a mouse infection model, demonstrating that proteasomal degradation and pupylation support Mtb survival in the host. It was hypothesized that the proteasomal degradation pathway might be involved in removal of proteins damaged by RNI and ROS. The chemical effects of both RNI and ROS and the nonspecific damage they cause to proteins, lipids and DNA will be discussed in more detail in a later section of this review. However, the connection between the PPS locus and NO stress turned out to be more complex, affecting several specific pathways, which will be the focus in this section of the review.

One mechanism was identified for the PPS locus in Mtb through a suppressor mutagenesis screen of NO sensitivity (Samanovic et al., 2015). The screen showed that disruption of a gene with homology to a plant enzyme involved in cytokinin biosynthesis called “lonely guy (LOG)” could reverse the NO-hypersensitive phenotype of the *mpa* deletion strain, suggesting that increased cytokinin production during infection was responsible for the observed phenotype. Plant LOG enzymes possess cytokinin-specific phosphoribohydrolase activity, cleaving the inactive cytokinin nucleotides to release the active free-base cytokinins (Kurakawa et al., 2007). Indeed, Mtb Log (Rv1205) produces cytokinins in Mtb and is a pupylation target. It accumulates in the *mpa* deletion strain as well as in a mutant strain where the target lysine is changed to alanine, indicating that Log is degraded by the Mpa-proteasome in a pupylation-dependent manner (Samanovic et al., 2015) (Table 1).

**TABLE 1:** Overview of the involvement of the 20S proteasome in select stress conditions in various Actinobacteria described in this review.

Stress Condition	Involvement of proteasome or pupylation	Substrates	Organism	References
Oxidative stress	Regulation of differentiation and resistance	Unknown	*M. tuberculosis*	Darwin et al. (2003); De Mot et al. (2007); Boubakri et al. (2015); Compton et al. (2015)
			*S. coelicolor*	
Reactive nitrogen intermediates (NO)	Removal of cytokinin producing enzyme (cytokinin breakdown products sensitize Mtb to NO)	Cytokinin synthesis enzyme Log	*M. tuberculosis*	Darwin et al. (2003); Pearce et al. (2006); Samanovic et al. (2015); Samanovic et al. (2018)
Nitrogen limitation	Recycling of amino acids for biogenesis; folding of proteins involved in nitrogen assimilation	Multiple proteins involved in nitrogen metabolism and assimilation (ex. GlnA, HrcA)	*M. smegmatis*	Becker et al. (2019); Fascellaro et al. (2016)
			*M. tuberculosis*	
DNA damage	Transcription activation of DNA repair genes and pupylation of DNA repair proteins during recovery	Broad range of DNA repair proteins (ex. RecA)	*M. smegmatis*	Olivencia et al. (2017); Müller et al. (2018); Müller et al. (2019)
Iron limitation	Release of iron storage	Ftn	*C. glutamicum*	Küberl et al. (2016); Küberl et al. (2014)
Copper homeostasis	Involvement in regulation of RicR regulon (ex. MymT and MmcO)	Unknown	*M. tuberculosis*	Festa et al. (2011); Shi et al. (2014)
Heat shock	ATP- and pupylation-independent degradation of HspR to allow chaperone expression (e.g. DnaK and ClpB)	HspR	*M. tuberculosis*	Jastrab et al. (2015); Jastrab et al. (2017)
Carbon starvation	Unknown	Unknown	*M. smegmatis*	Ziemski et al. (2018)

Cytokinins are adenine derivatives with substitutions at the amino group at C6 of the purine ring. In Mtb, isoprenoid cytokinins like N6-(Δ2-isopentenyl)adenine (iP) or 2-methylthio-iP are the most abundant. While it is well-established that in plants cytokinins act as hormones influencing cell growth and differentiation (Mok and Mok, 2001), their role in bacteria and specifically in Mtb is less well understood. In a follow-up study to the suppressor screen, the authors observed cytokinin-induced upregulation of a protein of unknown function (Rv0077c) that resulted in loss of acid-fast staining of Mtb, hinting at a possible remodeling of components in the cell envelope (Samanovic et al., 2018). They could show that Rv0077c is repressed by neighboring TetR-like repressor Rv0078 in absence of cytokinins. However, in the mouse model of tuberculosis, constitutive expression of Rv0077c by disruption of the repressor gene did not affect virulence, leaving it unclear in which phase of infection and in what way the bacterium benefits from the upregulation. Interestingly, Rv0077c was reported as a putative pupylation substrate in a proteomic study previously, suggesting that the PPS might be involved in its removal after upregulation (Festa et al., 2010).

Regardless of their biological role in Mtb, the fact that production of cytokinins can render the bacterium sensitive to NO appears to be due to breakdown of cytokinins into aldehydes, which are rendered toxic in additional presence of NO (Samanovic et al., 2015). Pup-dependent degradation of Log prevents the cytokinin levels from overshooting and thereby supports survival during infection. This finding shows that the degradation of a specific pupylation substrate, in this case Log, can contribute significantly to the NO-sensitive phenotype of Mtb. Homologs of Log are also found in *M. smegmatis* (Msm) (MSMEG_5087), *M. bovis* (Mb1237), and *M. marinum* (MMAR_4233), suggesting that other mycobacteria also might produce cytokinins under certain conditions.

In eukaryotes, it is known that the proteasome is essential to recycle amino acids and hence contributes to nutrient homeostasis in the cell (Vabulas and Hartl, 2005; Suraweera et al., 2012). A similar role was proposed for the PPS in Msm under nitrogen starvation. An Msm strain deficient in *pup* and the proteasomal subunit genes exhibited a significantly more severe growth defect upon nitrogen limitation than the parent strain (Elharar et al., 2014). Interestingly, during the onset of nitrogen limitation (24 h) an increase in the levels of pupylated proteins was observed, but they were almost completely degraded a week into the starvation stress. Furthermore, probing of PPS member levels indicated that nitrogen starvation in Msm induces oscillations in their abundance for the duration of the starvation stress (Elharar et al., 2014), an expression phenotype that could not be observed under similar conditions for Mtb (Becker et al., 2019). The authors hypothesize that during nitrogen starvation the PPS takes the role of an amino acid recycling pathway to provide the bacterium with the required protein building blocks in times when amino acids cannot be obtained by *de novo* synthesis or from the environment.

Another study proposed that the PPS might also play a more specific role during nitrogen starvation in Msm by adjusting the levels of enzymes involved in nitrogen assimilation pathways (Fascellaro et al., 2016). Msm encodes a particularly high number of nitrogen related genes involved in nitrogen uptake, assimilation and regulation (Amon et al., 2009). The transcriptional regulator GlnR is the global nitrogen response regulator in Msm, controlling primary nitrogen metabolism and the switch to nitrate or urea as alternative nitrogen sources (Jenkins et al., 2013). Proteome analysis of an Msm *pup* deletion strain under nitrogen starvation revealed that levels of 17 proteins of nitrogen metabolism were altered compared to the parent strain under the same starvation stress, 9 of them members of the GlnR regulon. Interestingly, the nitrite reductase, regulator GlnR, as well as GlnR-regulated proteins (e.g. glutamine synthetase GlnA1) were less abundant in the *pup* deletion strain, thereby precluding straightforward Pup-mediated degradation. Furthermore, for some of them, lower mRNA levels were observed when pupylation was absent. Irrespective of the exact mechanism of regulation, these proteins play an important role for nitrogen assimilation when nutrients are limited and the PPS is involved in their regulation.

The nature of this involvement was elucidated for nitrate assimilation of Mtb (Becker et al., 2019). It was observed that Mtb strains deficient in either the *mpa, pafA* or *prcBA* genes could no longer grow on nitrate as a nitrogen source and secreted large amounts of nitrite, suggesting that the nitrate assimilation pathway was impaired at the level of the nitrite reductase complex NirBD, leading to accumulation of toxic levels of nitrite. A suppressor screen aimed at restoring the ability of the *mpa* mutant strain to assimilate nitrate, identified HrcA as a suppressor gene (Becker et al., 2019). HrcA is a transcriptional regulator that represses the chaperonin system genes *groES* (Rv3418c), *groEL1* (Rv3417c) and *groEL2* (Rv0440), and the gene *Rv0991c* (Stewart et al., 2002b). The authors could show that HrcA is a pupylation target *in vitro* (Becker et al., 2019). The fact that *groEL2* is suppressed in the *mpa* mutant strain is thus a direct consequence of impaired proteasomal degradation of HrcA. Client proteins of GroELS chaperonins require its function in order to gain their natively folded, active state (Horwich et al., 1993; Kong et al., 1993; Kumar et al., 2015; Horwich and Fenton, 2020). The strongly diminished nitrite reduction activity in the *mpa* mutant strain suggests that NirBD is functionally impaired due to deregulated GroELS levels. The screen also identified mutations in an essential gene that encodes the enzyme catalyzing the committed step in NAD biosynthesis (*nadD*). These turned out to be gain-of-function mutations boosting NAD levels and thereby supporting NirBD activity, which requires the presence of adequate levels of NAD^+^/NADH.

These findings illustrate that the PPS gene locus also influences nitrogen metabolic networks by indirectly affecting transcriptional regulation of a quality control pathway. The uncovered link likely also contributes to the observation that silencing of the *prcBA* genes in Mtb leads to lowered resistance against sodium nitrite and lowered persistence in mice (Gandotra et al., 2007). The effect of the PPS on nitrogen metabolism and resistance to nitric oxide stress is multifaceted and complex, highlighting the prominent role that pupylation and proteasomal degradation plays in nitrogen homeostasis.

### The Pup-Proteasome Gene Locus and the DNA Damage Response

Stable transmission of genetic information from one generation to the next is crucial for all living organisms. Although some level of mutagenesis provides the genetic diversity that allows bacteria to evolve, global DNA damage that would reduce their fitness and threaten survival must be dealt with swiftly. Mycobacteria, for example, are frequently exposed to conditions that can damage their genetic material, like macrophage-generated reactive oxygen species and reactive nitrogen intermediates experienced by pathogenic members inside the host (Weiss and Schaible, 2015), and UV exposure, metabolic endogenous oxidative or nitrosative stress or DNA damaging chemicals produced by other microorganisms for free-living mycobacteria.

The first indication that the PPS gene locus plays a role in the mycobacterial DNA damage response came from the observation that the levels of SOS response regulator, recombinase A (RecA), are significantly reduced in an Msm *pafBC* deletion strain (Olivencia et al., 2017) (Table 1). Although in mycobacteria, the homologous PafB and PafC proteins are encoded in an operon together with Pup ligase PafA (Festa et al., 2007; Olivencia et al., 2017), it was shown early on, that they are not required for degradation of proteasomal substrates (Festa et al., 2007). Their predicted N-terminal winged helix-turn-helix domains suggested that they might be involved in transcriptional regulation. Indeed, it was shown that *recA* transcript levels are decreased in a Δ*pafBC* strain compared to wild type and can be restored by complementation with *pafBC* (Olivencia et al., 2017).

Since its discovery about 40 years ago, the LexA/RecA-mediated SOS response had been considered the main regulation pathway of the bacterial DNA damage response (Radman, 1975; Little and Mount, 1982; Shinagawa, 1996). Under conditions where the bacteria do not experience DNA damage stress, DNA repair genes preceded by the so-called SOS box sequence are repressed by transcriptional repressor LexA. Single-stranded DNA (ssDNA) fragments occurring as a consequence of DNA damage, trigger derepression of these genes by a mechanism involving RecA, where RecA and ssDNA form a nucleoprotein filament that binds to LexA, triggering its autocatalytic cleavage and dissociation from the SOS box (Galletto et al., 2006; Giese et al., 2008; Butala et al., 2011). As it is also an important enzyme in the process of homologous recombination, RecA plays a dual role during DNA damage stress, namely as stress sensor and as a repair enzyme.

**FIGURE 3:**
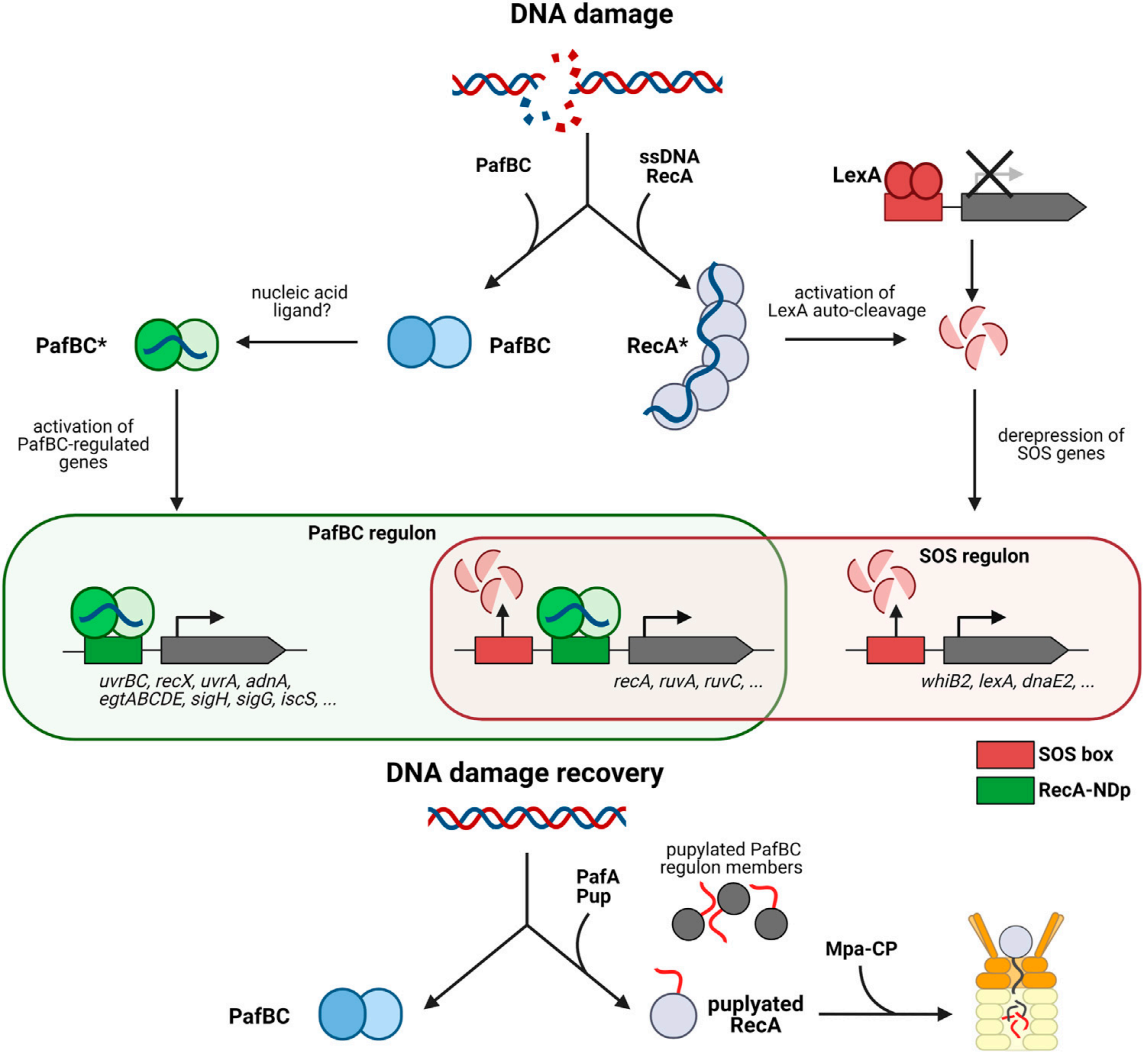
The pupylation locus in involved in the mycobacterial DNA damage response. The mycobacterial DNA damage response is mediated by two pathways, the SOS response mediated by repressor LexA and the response mediated by activator PafBC. The canonical SOS response pathway is illustrated on the right (LexA/RecA-dependent pathway). Upon DNA damage RecA forms filaments on ssDNA, which stimulates auto-cleavage of the repressor LexA. LexA dissociates from the SOS box which in turn, upregulates transcription of DNA damage response genes such as *recA*. The left side shows the PafBC-dependent DNA damage response pathway. Upon binding of a response-producing ligand (hypothesized to be a nucleic acid) PafBC is activated and binds to the RecA-NDp promoter, activating the transcription of many genes involved in DNA repair and oxidative stress response. Upon return to normal conditions, RecA along with several other PafBC regulon members (EgtD, TopoN, IscS, RuvA, etc.) are pupylated and removed by the Pup-proteasome system to recover fully from DNA damage. Interestingly, some genes such as *recA*, *uvrA*, and *uvrC* are regulated by the PafBC regulon as well as the SOS regulon.

Mycobacteria also possess this canonical repression/release mechanism *via* LexA and RecA, and by affecting RecA levels PafBC impacts the SOS response (Figure 3). In Mtb, roughly 25 genes were reported to be under LexA control, amongst them also *recA* itself (Smollett et al., 2012). However, it was realized early on that the majority of inducible DNA repair genes in Mtb can still be induced in the absence of the *recA* gene, suggesting that a LexA/RecA-independent pathway must exist (Davis et al., 2002; Rand et al., 2003). In fact, based on the available transcriptomic data, it was even possible to deduce a consensus motif for this hypothetical additional pathway, aptly named RecA-NDp (RecA-independent promoter) based on the fact it is not regulated by the canonical pathway (Gamulin et al., 2004). Furthermore, the RecA-NDp promoter is not restricted to mycobacteria but extends to other Actinobacteria. The nature of regulation of this pathway and the identity of the regulator, however, remained unknown.

It was only recently that a fuller picture began to emerge. A combination of transcriptomics and genome-wide PafBC binding site analysis in Msm revealed that PafBC acts as a global transcriptional activator, controlling a regulon of more than 150 genes in response to the DNA damaging agent mitomycin C (Müller et al., 2018) (Figure 3). Members of the regulon include many proteins involved in DNA replication, recombination and repair, like for example the UvrABC nucleotide excision repair complex, the two main end resectioning complexes RecBCD and AdnAB, important for homologous recombination, along with strand exchange mediating RecA and holiday junction binding protein RuvA and resolvase RuvC. Sigma factor H shown to be activated in Mtb upon heat stress and oxidative stress (Sharp et al., 2016), is also upregulated by PafBC (Müller et al., 2018). Likewise the gene cluster for the biosynthesis of the protective, redox-active compound ergothioneine is present in the regulon. This suggests that PafBC is also important for the oxidative stress response discussed in the next section of this review.

Interestingly, the consensus sequence motif identified for PafBC binding closely resembles the RecA-NDp motif. These results not only established PafBC as the elusive regulator of the LexA/RecA-independent DNA damage response pathway, but furthermore demonstrated that transcriptional activation in addition to repression-release is involved in the transcriptional response to DNA stress (Müller et al., 2018). Among the members in the regulon are several genes that feature both the RecA-NDp as well as the SOS box in their upstream regions and are thus regulated by both the PafBC-dependent activating branch and the LexA/RecA-repression-release branch of the DNA damage response. RecA itself is regulated by both pathways, indicating that PafBC also indirectly influences the SOS response and that there exists tight cooperation between the two pathways (Figure 3).

The study also provided a functional link to the PPS, explaining the association of PafBC with that gene locus. Determination of the pupylated proteome present in Msm exposed to mitomycin C-induced DNA stress identified 26 PafBC regulon member proteins as pupylation targets, including RecA (Müller et al., 2018). Analysis of the RecA protein levels during mitomycin C exposure and in the recovery phase after removal of the DNA damaging reagent, showed that only wild type Msm but not Msm strains deficient in pupylation or proteasomal degradation were able to return RecA to pre-stress levels (Müller et al., 2018). This demonstrates that the PPS is required to ensure a temporally controlled, transient DNA stress response.

Although PafBC activates its regulon members specifically under DNA stress, the mRNA and protein levels of PafBC remain unchanged upon DNA stress exposure, indicating that a response-producing ligand is most likely involved (Olivencia et al., 2017; Müller et al., 2018). Based on its primary sequence, PafBC was classified with a family of bacterial regulators containing a so-called WYL domain, a domain of unknown function named for a conserved, consecutive Trp-Tyr-Leu sequence motif. Determination of the crystal structure of a naturally fused PafBC ortholog from *Arthrobacter aurescens* revealed that the N-terminal winged helix-turn-helix domain is followed by a domain containing the conserved WYL motif and featuring an Sm-fold (Müller et al., 2019), frequently encountered in RNA-binding proteins like for example the bacterial RNA chaperone Hfq (host factor for RNA bacteriophage Qβ replication) (Khusial et al., 2005; Updegrove et al., 2016). In Hfq, a highly conserved loop in the Sm-2 region makes contact to the backbone of its RNA ligands (Schumacher et al., 2002; Khusial et al., 2005). Mutation of two arginine residues in the structurally homologous loop in *pafBC* renders it unable to rescue the mitomycin C-sensitive phenotype of the *pafBC* deletion strain (Müller et al., 2019). These results led to the suggestion that the proposed response-producing ligand is a nucleic acid molecule. Interestingly, bioinformatic analysis showed that transcriptional regulators featuring a winged helix-turn-helix domain followed by a WYL domain occur widely in bacteria, but not in eukaryotes (Müller et al., 2018). Indeed, it is likely that all WYL domain containing transcriptional regulators are activated according to a similar mechanism as PafBC.

The PafBC-mediated DNA damage response also plays a role for the action of fluoroquinolone antibiotics, which are important second-line drugs for treating multi-drug resistance tuberculosis infections. Fluoroquinolones target DNA gyrase and topoisomerase IV, causing the release of DNA with single or double strand breaks (Drlica et al., 2008). It was shown that a *pafC* deletion strain of Msm is strongly sensitized toward fluoroquinolone antibiotics (Li et al., 2015). This finding makes sense in light of the fact that PafBC induces DNA repair genes involved in double-strand break repair (Müller et al., 2018). The fact that WYL-domain containing transcriptional regulators appear to be restricted to the bacterial kingdom (Müller et al., 2018), renders PafBC an attractive drug target, since PafBC inhibitors could be administered in combination with fluoroquinolone antibiotics to escalate their effect.

### The Pup-Proteasome System Supports Actinobacterial Survival Under Oxidative Stress

Although the evolutionary origin of Actinobacteria predates oxygenation of the atmosphere (Battistuzzi et al., 2004), the majority of modern Actinobacteria are aerobic, where molecular oxygen serves as the final electron acceptor of the respiratory chain (Barka et al., 2016). Aerobic respiration has the advantage of high energy efficiency, however, partially reduced oxygen species occur as byproducts of aerobic metabolic activity. Endogenous production of superoxide (O_2_^−^) and hydrogen peroxide (H_2_O_2_) is largely due to autoxidation of flavoenzymes by transfer of electrons from the flavin cofactor to molecular oxygen (Imlay, 2003). In the presence of ferrous iron (Fe^2+^), which is formed in the cellular environment from Fe^3+^ by reaction with FADH_2_ or cysteine as reductant, hydrogen peroxide can react to from hydroxyl radicals according to the Fenton reaction (Fenton, 1894). These reactive oxygen species (ROS) can cause DNA damage and protein modifications leading to loss of function (Cabiscol et al., 2000; Farout and Friguet, 2006). For this reason, bacteria are armed with detoxifying enzymes such as catalases, peroxiredoxins, and superoxide dismutases to combat these harmful agents. Mutants lacking these ROS detoxifying enzymes exhibit growth defects even under standard conditions, as ROS are continuously formed inside the cell as side products of metabolic reactions under aerobic conditions (Carlioz and Touati, 1986; Seaver and Imlay, 2001). In addition, bacteria may experience exogenous oxidative stress from their surrounding environment. Therefore, transcriptional and post-translational regulation mechanisms exist to respond to different levels of oxidative stress, controlling generation of protective redox molecules and expression of defense proteins or repair enzymes.

Eukaryotic proteasomes were shown early on to be involved in the removal of oxidized proteins (Farout and Friguet, 2006). Bacterial pupylation and proteasomal degradation has also been linked to counteracting oxidative stress. In this section, we will discuss where and when Actinobacteria encounter oxidative stress and how the PPS and PafBC regulon are involved in the oxidative stress response.

One major host defense strategy is the generation of ROS, reactive nitrogen species (RNS), and reactive chlorine species (RCS), which pathogenic bacteria must simultaneously contend with upon entering the host. A well-known example for such a hostile microenvironment are macrophages that are colonized by Mtb (Winterbourn et al., 2006; Flannagan et al., 2015). As mentioned previously in this review, a transposon mutagenesis screen in Mtb identified members of the PPS as being involved in RNS resistance (Darwin et al., 2003), linking the PPS to RNS stress before the pupylation pathway had been discovered. In the study, RNS stress was induced by exposing Mtb to NaNO_2_ at an acidic pH of 5.5 which led to significant survival reduction of Mtb lacking PPS components in comparison to wild type Mtb, while no phenotype was observed for these deletion strains under standard laboratory conditions (Darwin et al., 2003). Furthermore, it was shown in the mouse infection model that Mtb requires the proteasome to persist after infection (Gandotra et al., 2007). These studies established the PPS locus as relevant for survival of Mtb in the host and stimulated an interest in the proteasome as a drug target (Totaro et al., 2017).

An earlier study aimed at determining the protein targets of nitrosative stress identified 29 S-nitrosylated proteins in Mtb exposed to sodium nitrite (Rhee et al., 2005), 24 of which were later also identified in studies determining the pupylated proteomes of Mtb or Msm (Festa et al., 2010; Poulsen et al., 2010; Watrous et al., 2010). Although this overlap is interesting considering one of the first stress conditions linked to the PPS was its protective effect against nitrosative stress (Darwin et al., 2003), it must be taken with caution, since no causal connection was made between nitrosylation and proteasomal degradation and since for none of those proteins an impaired function has been reported upon nitrosylation.

Curiously, the same study found that the Mtb PPS mutant strains were more resistant to hydrogen peroxide, a phenotype also observed in another study upon silencing of the proteasomal subunit genes (De Mot et al., 2007). Apart from Mtb, the PPS has also been investigated under different oxidative stresses in other Actinobacteria. A proteome analysis in *S. coelicolor* revealed the accumulation and depletion of proteins in mutants lacking ARC, Dop, Pup or the proteasome, respectively (De Mot et al., 2007). Interestingly, the mutants show increased resistance to cumene hydroperoxide that coincides with the accumulation of haloperoxidase SCO0465 in all of the mutants which could explain the hyper resistance of the mutants. Notably, hyper resistance was not observed for the mutant lacking ARC if oxidative stress was induced with diamide or plumbagin while the *dop*, *pup*, and proteasome deficient strains show similar hyper resistance. In 2015 two independent studies showed that genetic deletion of *pup* leads to H_2_O_2_ hypersensitivity in *S. coelicolor*. One study observed that the H_2_O_2_ tolerance of the strain disrupted in *prcB* was comparable to that of the wild type strain rather than the *pup* knockout strain, which might indicate a proteasome-independent role of pupylation in oxidative stress defense in *S. coelicolor* (Boubakri et al., 2015). In contrast, the other study reported H_2_O_2_ hypersensitive phenotypes for *Δprc* (SCO1643–1644), *Δpps* (SCO1643–1646, lacking Pup and the proteasomal subunit genes) and *ΔpafA* (SCO1640) strains (Compton et al., 2015). However, their *pafA* knockout exhibits a sporulation defect while their *prc* and *pps* knockouts do not, which would support a role of pupylation acting independent of proteasomal degradation in this specific context (Compton et al., 2015). In those studies, the exact mechanism of how pupylation contributes to overcoming oxidative stress in *S. coelicolor* remains unclear.

Besides DNA damage repair pathway genes, the PafBC regulon comprises genes involved in the oxidative stress response. As mentioned in the previous section on the DNA damage response, this includes the ergothioneine biosynthesis gene cluster *egtABCDE* that encodes a secreted antioxidant low molecular weight thiol (Müller et al., 2018). Ergothioneine scavenges hydroxyl radicals and detoxifies peroxynitrite due to its high redox potential (Akanmu et al., 1991; Cumming et al., 2018). Hence, ergothioneine is essential for Mtb survival in macrophages because of its protective properties against oxidative and nitrosative stress (Richard-Greenblatt et al., 2015). Furthermore, ergothioneine acts as metal chelator blocking copper-induced oxidation of DNA (Zhu et al., 2011). Interestingly, the PPS also appears to be involved in shutting down the upregulation of ergothioneine biosynthesis, as EgtC and EgtD were identified as pupylation substrates during DNA stress (Müller et al., 2018). In addition to the ergothioneine gene cluster, two sigma factors involved in stress responses are contained in the PafBC regulon; SigH is activated upon heat shock, oxidative stress, and nitric oxide stress and induces transcription of thioredoxin, methionine sulfoxide reductase and chaperones such as Hsp70/DnaK (Sharp et al., 2016). The other sigma factor in the PafBC regulon is SigG which is known to play a role in Mtb during infection of host macrophages, however, its regulon is less well understood (Cappelli et al., 2006; Gaudion et al., 2013). Another protein of the PafBC regulon, the IscS-like cysteine desulfurase, removes sulfur from cysteine to produce alanine and a thiol group required for Fe-S core formation and is also involved in the oxidative stress response (Rybniker et al., 2014).

Taken together, the studies in mycobacteria and other Actinobacteria show that the PPS gene locus supports bacteria under oxidative stress, that the observed phenotypes are multicausal and that there are facets of the roles played by the PPS under oxidative conditions that remain to be discovered.

### Role of Pupylation in Actinobacterial Metal Homeostasis

Many enzymes involved in fundamental biological processes require metals as cofactors for their catalytic activity (Andreini et al., 2008). In fact, such metalloenzymes constitute about one-third of all known enzymes (Holm et al., 1996). Iron ions are amongst the most abundant cofactors and found in a wide variety of enzymes playing a role in amino acid and pyrimidine biogenesis, the tricarboxylic acid cycle, electron transport, oxygen sensing and transport, as well as nucleic acid synthesis (Barton et al., 2007; Silva-Gomes et al., 2013). Therefore, iron is pivotal for almost all living organisms with only few exceptions (Posey and Gherardini, 2000). Iron ions exist in one of two redox states under physiological conditions: the reduced, highly water-soluble Fe^2+^ ferrous form is capable of forming toxic radicals and is predominantly found under anaerobic conditions and at low pH; the oxidized, highly insoluble Fe^3+^ ferric form that is non-toxic and most prevalent under aerobic conditions. Iron homeostasis in the cell is tightly regulated and excess iron ions in the cytosol are stored as ferric oxide inside the iron storage protein bacterioferritin, a homo-24-meric cage able to hold up to 4,500 iron atoms (Andrews, 1998; Kurthkoti et al., 2015). Iron ions can be released from bacterioferritin when free iron levels become limiting to ensure that the enzymatic processes requiring this cofactor can be supported. In addition to the release of stored iron, Actinobacteria have evolved specialized mechanisms to overcome iron limitation; for example the secretion of iron chelators, so-called siderophores, that scavenge iron from the environment (Wang et al., 2014), which is for example crucial during Mtb infection (Rodriguez and Smith, 2006). The mechanisms of iron acquisition by siderophores and other iron uptake mechanisms such as heme uptake through hemophores, sequestration of holo-transferrin and holo-lactoferrin, as well as iron diffusion through low-affinity porins have been extensively reviewed (Banerjee et al., 2011; Ratledge, 2013; Fang et al., 2015; Chao et al., 2019). Here, we will describe the role of pupylation in iron homeostasis in Actinobacteria.

A study in the soil-based Gram-positive actinobacterium *Corynebacterium glutamicum* showed that mobilization of iron stores under iron limitation is dependent on pupylation (Küberl et al., 2016). Bacterioferritin Ftn was identified as a pupylation target and *C. glutamicum* *pup, pafA* and *dop* knockout strains showed growth defects in iron-limited medium (Küberl et al., 2014; Küberl et al., 2016). Interestingly, *C. glutamicum* lacks the proteasomal subunits *prcBA* which indicates degradation-independent iron release from Ftn. The authors propose a mechanism in which pupylated Ftn is unfolded by ARC leading to disassembly of the 24-mer and iron release. Monomeric Ftn is then recycled by Dop and can enter a new cycle of oligomerization and storage of iron. In addition, microarrays showed that mRNA levels of iron-dependent proteins were significantly depleted in the *pup* knockout strain suggesting that pupylation of Ftn is also indirectly involved in other aspects of iron homeostasis. It is still unclear how many Ftn subunits need to be pupylated for successful disassembly and how iron is solubilized from the mineral core (Küberl et al., 2016).

This study is a prime example of pupylation and unfolding in cellular homeostasis in the absence of the 20S proteasome. Nevertheless, the sensing trigger for pupylation of Ftn in *C. glutamicum* is not known to date. The study also raises the question about potential other proteasome-independent roles of pupylation. According to a pupylome study performed in Msm, BfrB is pupylated at the conserved lysine residue K10 (Watrous et al., 2010). Pupylation of BfrB could not be detected in Mtb by mass-spectrometry, but is observed in two independent pupylomes in Msm (Festa et al., 2010; Poulsen et al., 2010). This might suggest the Ftn homolog BfrB in mycobacteria could be a degradation substrate. However, this has not been demonstrated directly and it remains possible that Mpa can act on its own as in *C. glutamicum*.

Copper is another essential micronutrient in living organisms required for activity of multiple enzymes involved in electron transport, denitrification and oxidative respiration (Tavares et al., 2006). Yet, copper homeostasis needs to be tightly regulated since copper is toxic in high concentrations due to ROS generation (Dennison et al., 2018). Interestingly, one of the macrophage defense mechanisms is the accumulation of copper within the phagosome upon mycobacterial infection (Wagner et al., 2006). The PPS was linked to copper homeostasis for the first time through a transcriptional screen that compared the Mtb mutant strains disrupted in the gene coding for the Pup ligase (*pafA*) or the gene coding for the proteasomal ATPase (*mpa*) with wild type Mtb (Festa et al., 2011). Of 4009 predicted open reading frames fewer than 2% of the genes showed differential expression in the *pafA* and *mpa* disrupted strains compared to the wild type. One of those genes is the copper sensing repressor RicR that contributes to virulence of Mtb (Festa et al., 2011; Shi et al., 2014). Interestingly, RicR transcript levels are downregulated in *pafA* and *mpa* disrupted strains compared to wild type Mtb. However, RicR does not accumulate in pupylation deficient strains under all tested growth conditions so far (Festa et al., 2011) and has not been identified in any of the pupylomes. The authors hypothesize that transcriptional RicR downregulation in the pupylation deficient strains might be a downstream result of the accumulation of copper-binding proteins that are pupylation substrates. In turn, accumulation of copper-binding proteins might mimic copper limiting conditions that lead to RicR repression (Festa et al., 2011). However, pupylation candidates leading to the potential downstream event of RicR repression are currently lacking.

## Contribution of Pupylation-Independent Proteasomal Degradation to Actinobacterial Stress Responses

Bacterial stress responses are as diverse as the environmental insults that threaten the survival of the bacteria. One environmental parameter that has the ability to affect a multitude of cellular processes simultaneously is high temperature. Proteins mediate the majority of cellular reactions and pathways and as such affect essentially every aspect of cellular function. Their individual activity is supported not only by their primary sequence, but also by the precise three-dimensional structures they adopt and the complexes they form. When ambient temperature suddenly increases, cellular proteins can misfold, adopt inactive conformations or aggregate, leading to loss of function and threatening survival (Lewis and Pelham, 1985; Pelham, 1986). Thus, bacteria are equipped with intricate regulatory mechanisms to induce the expression of heat shock chaperones that are able to promote folding during heat stress as a way to quickly adapt to this challenging environment (Lewis and Pelham, 1985; Pelham, 1986; Hartl, 1996). Two major chaperone machineries in the bacterial cytosol are the GroEL/GroES and the DnaKJ/GrpE chaperone systems that are under positive control by sigma factors in *E. coli*, yet negatively regulated in several Gram-positive bacteria including Streptomyces and in mycobacteria (Bukau and Horwich, 1998; Narberhaus, 1999; Stewart et al., 2001; Bucca et al., 2003). GroEL and DnaK belong to the Hsp60 and Hsp70 family, respectively (Horwich and Fenton, 2020). In Mtb, there are two *groEL* loci (Rv0440 and Rv3417c), both controlled by the repressor HrcA (Stewart et al., 2002b), which is a pupylation substrate, hence linking the chaperone system to the PPS as discussed already in a previous section of this review.

**FIGURE 4:**
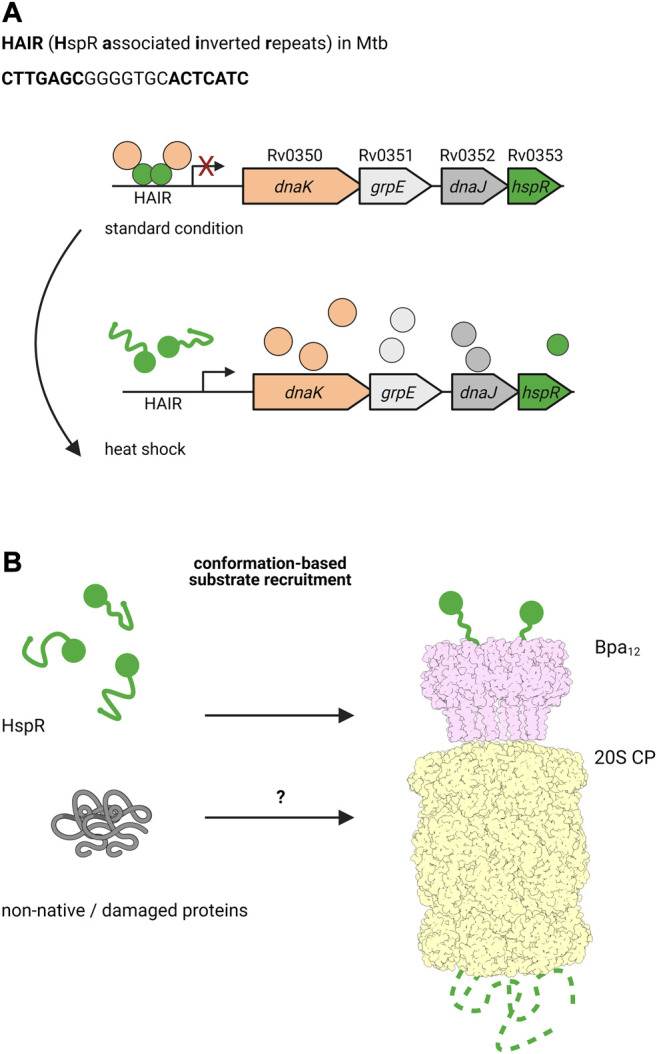
The ATP-independent proteasomal activator Bpa plays a role during heat shock in Mtb. **(A)** Under standard conditions, the heat shock chaperone DnaK co-represses the transcription of the *dnaKJgrpE-hspR* operon together with the transcriptional regulator HspR, which binds the HAIR motif operator. Upon heat shock, HspR is partially denatured and dissociates off the promoter to allow transcription of *dnaK*, *grpE*, *dnaJ* and *hspR*, ultimately leading to production of the encoded proteins. **(B)** HspR (green) is a substrate for the ATP- and pupylation-independent proteasomal degradation facilitated by Bpa (light pink). Bpa assembles into a homo-dodecameric ring and interacts with the 20S proteasome (light yellow) by inserting its C-terminal GQYL motif into binding pockets between the α-subunits of the core particle. Though there is no overall homology to Mpa/ARC, the GQYL motif is shared between both proteasomal activators.

It has been shown that the alternative degradation complex, formed by the bacterial proteasome with ATP-independent, ring-shaped activator Bpa, is involved in regulation of the DnaK operon in Mtb (Jastrab et al., 2015; Jastrab et al., 2017). In mycobacteria, transcription of *dnaK* is controlled by heat shock repressor HspR (Stewart et al., 2002b; Stewart et al., 2001; Stewart et al., 2002a). Under normal conditions, HspR binds to its operator, the HAIR motif (HspR associated inverted repeats), to repress the expression of *dnaK*, its co-chaperone *dnaJ*, the exchange factor *grpE* and *hspR* itself (Bucca et al., 1995; Bucca et al., 1997). Interestingly, DnaK binds HspR acting as co-repressor, and upon heat shock, the complex detaches to allow the expression of these heat shock chaperones (Figure 4) (Bucca et al., 2000; Bandyopadhyay et al., 2012; Parijat and Batra, 2015). In addition to DnaK and the other proteins expressed in the *dnaKJEhspR* operon, HspR also regulates the expression of chaperone genes *clpB* and *acr2*, which belong to the Hsp100 and Hsp20 family, respectively (Grandvalet et al., 1999). HspR is one of the few known proteasomal substrates that is recruited by the pupylation-independent activator Bpa (also called PafE) for proteasomal degradation (Figure 4) (Jastrab et al., 2015).

Bpa was first identified by a full genome search in Mtb for genes that feature a C-terminal motif similar to the proteasome interaction motif found at the C-termini of Mpa (Delley et al., 2014). Though otherwise lacking any structural or sequence homology with Mpa, Bpa contains the same GQYL sequence at the C-terminus that in Mpa mediates binding to the α-subunits of the 20S proteasome. Bpa occurs in every actinobacterial species containing the 20S proteasome, but is absent in all Actinobacteria lacking the proteasomal subunits. Biochemical analysis demonstrated that Bpa forms a homooligomeric ring and is able to interact with the wild type 20S proteasome to degrade substrate in a pupylation and ATP-independent manner. Structural studies showed that Bpa, unlike the hexameric Mpa, forms a dodecameric ring with a funnel-like opening (Bolten et al., 2016; Hu et al., 2018).

ATP-independent activators were already known for the eukaryotic proteasome, where they were also shown to recruit substrates independent of ubiquitination. It has been hypothesized that disorder and low substrate stability may be substrate determinants, as supported by the degradation of the unstructured tau protein by the PA200 proteasomal activator in complex with the 20S proteasome *in vitro* (Huang et al., 2016). Similarly, it was shown that in bacteria, Bpa is able to facilitate the proteasomal degradation of the model substrate β-casein (Delley et al., 2014), which is used to mimic unstructured proteins due to its extended non-globular structure. This led to the hypothesis that upon stress induction, cytosolic proteins may become damaged or denatured, rendering them targets for Bpa-mediated proteasomal degradation. Indeed an Mtb *bpa* knockout strain showed a heat sensitive phenotype when grown at 45 °C (Jastrab et al., 2015). However, the authors also observed that *dnaK* and *clpB* mRNA levels dropped, suggesting that the phenotype is due to accumulating HspR that represses *dnaK* and *clpB* transcription to a higher extent. This in turn would have an impact on the quality control of the bacterium under stress. However, the two might not have to be mutually exclusive. Bpa might be involved in two aspects of the heat shock response: the regulation of heat shock response through proteasomal degradation of HspR and the removal of non-native proteins damaged during heat stress. Interestingly, loss of function mutations reversing the heat-sensitive phenotype of the *bpa* knockout strain were found in the HspR DNA binding domain (Jastrab et al., 2017). In addition to heat shock, it was suggested that Bpa also plays a vital role in Mtb virulence, as a *bpa* knockout strain was attenuated in mouse lungs and spleens (Jastrab et al., 2015). Hence, Bpa may play a role in other important stress responses that is yet to be discovered.

Although association with other ATP-dependent proteases could be preferred pathways to rid the cell of damaged or non-native substrates, it has been suggested that Bpa could have an important role under stressful conditions when ATP becomes limiting, for example during oxygen or nutrient limitation (Jastrab et al., 2017). Other stresses have yet to be tested and the heat shock sensitivity of a *bpa* knockout strain has only been seen in Mtb to date.

Recently, a new pupylation independent proteasomal interactor was identified (Ziemski et al., 2018). The Cdc48-like protein of Actinobacteria (Cpa) is a hexameric ATPase, like Mpa, and interacts with the wild type 20S proteasome *in vitro*. Cpa is homologous to the AAA protein Cdc48 in eukaryotes that, in coordination with the eukaryotic proteasome and various cofactors, is involved in multiple biological processes (Baek et al., 2013). Though best known for its involvement in ER-associated degradation (ERAD), eukaryotic Cdc48 plays vital roles in many other biological functions extensively reviewed elsewhere (Wolf and Stolz, 2012; Yamanaka et al., 2012; Baek et al., 2013).

Similar to Bpa, Cpa occurrence has only been shown in Actinobacteria that also harbor the genes for the α- and β-subunits of the 20S proteasome. Although Cpa competes with Mpa for binding to the 20S proteasome *in vitro*, no substrates have yet been found for Cpa-mediated proteasomal degradation. Notably, Cpa does not feature the conserved C-terminal GQYL interaction motif found both in Mpa and Bpa, and the interaction determinants are poorly understood (Ziemski et al., 2018).

The only information available to date on the role of Cpa comes from *in vivo* studies, which were carried out in Msm. In a *cpa* knockout strain in Msm, a mild phenotype was observed under carbon starvation, suggesting that Cpa may be involved in stress conditions where nutrients like carbon are limited. Bacteria in nature can often be starved of carbon, for example in marine environments where the carbon concentration is significantly lower or is in a bio-unavailable form (Morita, 1988). In the soil, carbon is also not always found in a state that can be readily incorporated (Lockwood, 1977).

Comparative proteomic analysis of the Msm *cpa* knockout showed significant accumulation of proteins involved in translation and ribosomal biogenesis. One possibility is that Cpa, in complex with the 20S proteasome, plays a role in disassembly and removal of ribosomal proteins under nutrient limited conditions. However, biochemical data is currently lacking to support this hypothesis and additional studies are required to understand the molecular basis of this phenotype. It is also possible that, like its eukaryotic counterpart Cdc48, Cpa is additionally involved in cellular pathways not dependent on proteasomal degradation.

## Concluding Remarks

The Pup-proteasome gene locus of Actinobacteria provides this large and diverse group of organisms with an advantage to grow and proliferate under the demanding and rapidly changing conditions they encounter in their natural surroundings. In all actinobacterial species investigated to date, phenotypes are observed under a variety of stress conditions but growth is normal or only very mildly affected under standard laboratory culture conditions. In complex with different ring-shaped activators, the 20S proteasome supports the survival of Actinobacteria in hostile conditions, including starvation, reactive nitrogen intermediates, oxidative stress, and heat shock.

Although these stresses on the surface appear to represent separate challenges and occur as a consequence of different events, they present an interwoven network of effects on various aspects of actinobacterial biology, and response mechanisms to one kind of stress also play a role during other experienced insults. The mechanistic complexity of PPS involvement in these stress responses is beginning to emerge. For example, oxidative stress due to ROS can cause irreversible protein modifications and the 20S proteasome is thought to be involved in removal of these aberrant proteins. However, oxidative stress can also lead to DNA damage through double stranded breaks, causing PafBC to activate the LexA/RecA-independent DNA damage response pathway. Furthermore, the two proteasomal degradation pathways, the pupylation-mediated and the Pup-independent pathway, can address the same stress from different directions. For example, the PPS is involved in the expression of two major chaperone machineries, the DnaKJGrpE and the GroELS chaperone systems, which are important for bacteria to adapt to stress like temperature shock. In complex with the ATP-independent activator Bpa that recruits substrates independent of pupylation, the 20S proteasome degrades the repressor HspR to allow for expression of the *dnaK* operon. On the other hand, HrcA responsible for repressing the *groEL1*, *groEL2*, and *groES* genes, is a pupylation substrate and is degraded by the 20S proteasome in complex with Mpa. In addition, GroEL is necessary for proper folding of nitrite reductase NirBD, linking the PPS to nitrogen metabolism and nitrosative stress. The versatility of the bacterial 20S proteasome, shown by its ability to interact with multiple activators to promote survival of Actinobacteria under different stresses, demonstrates that it plays an important role in the complex actinobacterial stress response and quality control pathways.

Given its involvement in various stress response pathways that are relevant to the survival of Mtb inside macrophages, the proteasome and other members of the PPS locus constitute attractive drug targets for treatment of Mtb infections. In fact, multiple inhibitors against the Mtb 20S proteasome have been designed and shown to make Mtb susceptible to its host’s immune system without heavily disrupting function of the eukaryotic proteasome (Totaro et al., 2017). With the emergence of multi-resistant and even completely resistant Mtb strains, new avenues to treat Mtb infections are urgently needed. Already today, combination therapies are usually used to treat Mtb patients. Drugs targeting the PPS could be another weapon in this arsenal, and could help to thwart the bacterium’s efforts to survive interventions by the host immune system and drug therapy.
